# Increased frequency of circulating CD19+CD24^hi^CD38^hi^ B cells with regulatory capacity in patients with Ankylosing spondylitis (AS) naïve for biological agents

**DOI:** 10.1371/journal.pone.0180726

**Published:** 2017-07-06

**Authors:** María-Belén Bautista-Caro, Eugenio de Miguel, Diana Peiteado, Chamaida Plasencia-Rodríguez, Alejandro Villalba, Irene Monjo-Henry, Amaya Puig-Kröger, Paloma Sánchez-Mateos, Emilio Martín-Mola, María-Eugenia Miranda-Carús

**Affiliations:** 1 Department of Rheumatology, Hospital Universitario La Paz-IdiPAZ, Madrid, Spain; 2 Laboratorio de Inmuno-Oncología, Hospital General Universitario Gregorio Marañón, Madrid, Spain; IMAGINE, FRANCE

## Abstract

Our objective was to study the frequency of circulating CD19+CD24^hi^CD38^hi^ B cells (Breg) in AS patients. To this end, peripheral blood was drawn from AS patients naïve for TNF blockers (AS/nb) (n = 42) and healthy controls (HC) (n = 42). Six patients donated blood for a second time, 6 months after initiating treatment with anti-TNFα drugs. After isolation by Ficoll-Hypaque, PBMCs were stained with antibodies to CD3, CD4, CD19, CD24, and CD38, and examined by cytometry. For functional studies, total CD19+ B cells were isolated from PBMCs of 3 HC by magnetical sorting. Breg-depleted CD19+ B cells were obtained after CD19+CD24^hi^CD38^hi^ B cells were removed from total CD19+ cells by cytometry. Total CD19+ B cells or Breg-depleted CD19+ B cells were established in culture and stimulated through their BCR. Secretion of IFNγ was determined by ELISA in culture supernatants. When compared with HC, AS/nb patients demonstrated a significantly increased frequency of Breg cells, which was independent of disease activity. Anti-TNFα drugs induced a significant reduction of circulating Breg numbers, which were no longer elevated after six months of treatment. Functional in vitro studies showed that the secretion of IFNγ was significantly higher in Breg-depleted as compared with total CD19+ B cells, indicating that Breg can downmodulate B cell pro-inflammatory cytokine secretion. In summary, an increased frequency of circulating CD19+CD24^hi^CD38^hi^ B cells is observed in AS/nb patients, that is not related with disease activity; anti-TNFα drugs are able to downmodulate circulating Breg numbers in AS.

## Introduction

The pathogenesis of Ankylosing spondylitis (AS), the prototype form of Spondyloarthritis (SpA), is not well understood, and evidence indicating a role for either autoinflammatory or autoimmune mechanisms has been described [1]. An interesting report by Cantaert et al [2] has shown an increased number of IL-10 producing CD19+CD5+ B lymphocytes in SpA.

B cells are not merely a source of antibodies [3]; they also act as very efficient antigen presenting cells and as cytokine producers [3]. In addition regulatory B cells, a subspecialized B cell subset, contribute to the maintenance of peripheral tolerance by downmodulating T and B cell function [4,5]. Phenotypical characterization of Bregs in mice or humans is not straightforward, and different definitions of Bregs have been proposed based on distinct cell surface markers [6]; however, Bregs have not been demonstrated to constitute a unique cell lineage [6]. Therefore, IL-10 production together with functional inhibition of T or B cell responses remain the gold standard for Breg definition [6].

In human peripheral blood, immature CD19+CD24^hi^CD38^hi^ B cells contain a high proportion of IL-10 producing cells [7,8] and functionally behave as suppressors of Th1 responses and Th17 differentiation [9]. In addition, CD19+CD24^hi^CD38^hi^ B cells are able to induce Treg and Tr1 phenotype from CD4+ T cells [9].

Patients with autoimmune conditions such as systemic lupus erythematosus (SLE) [7], RA [9], primary Sjögren′s syndrome [10], ANCA-associated vasculitis [11], and Systemic Sclerosis [12,13], have been shown to demonstrate altered numbers and/or function of circulating CD19+CD24^hi^CD38^hi^ B cells. In addition, whereas Cantaert et al described an increased number of circulating CD19+CD5+ B cells with a regulatory phenotype in SpA [2], Chen et al reported normal numbers of CD19+CD24+CD38+ B cells with decreased IL-10 production in AS patients [14]. To our knowledge, these are the two only published reports on the numbers of circulating B cells with regulatory properties in SpA. Therefore, our objective was to investigate on the frequency of circulating CD19+CD24^hi^CD38^hi^ B cells in AS and test the regulatory capacity of this B cell subset.

## Patients and methods

### Ethics statement

The study was approved by the Hospital La Paz—IdiPAZ Ethics Committee (protocol number HULP PI-883), and all subjects provided written informed consent according to the Declaration of Helsinki.

### Patients

Peripheral blood was obtained from 42 AS patients who had never received TNF blockers (AS/nb) (Table 1 and S1 Table) and from 42 age and gender-matched healthy controls (HC) (S2 Table). All subjects were studied between the years 2014 and 2016. Patients were recruited among those attending the outpatient Rheumatology Clinic at Hospital Universitario La Paz (Madrid, Spain). Inclusion criteria for patients were age greater than 18 years and AS diagnosis according to the 1984 modified New York criteria [15], exclusion criteria were a history of previous treatment with biological agents and infection with HBV, HCV or HIV. Healthy controls were recruited among hospital and laboratory workers. Inclusion criteria for controls were age greater than 18 years; exclusion criteria for controls were current or chronic medication intake, the presence of any known disease condition or infection with HBV, HCV or HIV. Forty-seven patients were approached and 42 accepted to participate; fifty controls were approached and 42 accepted to participate. There were no dropouts. Among patients, 28 were taking non-steroidal anti-inflammatory drugs (NSAIDs), 7 were receiving sulfasalazine (SSZ); 3 methotrexate (MTX) and 7 of them did not take any medication regularly. 31 patients had a pure axial disease (13 female, 18 male) and 11 patients (4 female, 7 male) had a combination of axial and peripheral manifestations (Table 1 and S1 Table). All subjects were of Western European descent.

**Table 1:** Clinical characteristics of AS/nb patients.

	AS/nb (n = 42)
Age (years); median (IQR)	52 (39.5–62)
Male; n° (%)	25 (59.5)
HLA-B27+; n° (%)	35 (85.4)
Duration of symptoms (yrs); median (IQR)	20.5 (10.0–34.5)
Time since diagnosis (yrs); median (IQR)	10 (4–19)
BASDAI; median (IQR)	4.6 (3.05–6.80)
BASFI; median (IQR)	2.9 (0.85–5.70)
ASDAS-ESR; median (IQR)	2.27 (1.92–3.38)
ASDAS-CRP; median (IQR)	2.80 (2.04–3.70)
CRP; median (IQR)	7.92 (3.25–12.45)
ESR; median (IQR)	9 (5.5–14.0)

AS/nb: AS patients naïve for TNF blockers

Six AS/nb patients intitiated treatment with anti-TNFα antibodies (2 infliximab, 2 golimumab, 1 adalimumab, 1 certolizumab). Four of them were male, and 5 were HLA B27+; age was 41.0 (33.0–59.5) years (median, IQR), duration of symptoms was 9.0 (2.0–19.5) years, time since diagnosis 6.0 (0.5–12.0) years, and ASDAS-CRP 3.87 (2.38–4.48). These patients donated blood on two occasions: right before the first dose of biological agent was administered, and 6 months thereafter. For each patient, one healthy subject served as control on both the first and second occasion.

### Isolation of B cells from human peripheral blood

Peripheral blood mononuclear cells (PBMCs) were separated immediately after blood sample collection, by Ficoll-Hypaque (GE Healthcare Biosciences AB, Uppsala, Sweden) density gradient centrifugation. Highly purified B cells were prepared from PBMCs by exhaustive immunomagnetic negative selection in an Automacs (Miltenyi Biotec, Bergisch Gladbach, Germany) using a B cell negative isolation kit (Miltenyi Biotec), containing biotinylated antibodies against CD2, CD14, CD16, CD36, CD43, and CD235a (glycophorin A). The purity of the isolated B cells as assessed by flow cytometry using a fluorochrome-conjugated anti-CD19 mAb was greater than 98%. Subsequently, Breg-depleted CD19+ B cells were obtained after removing CD19+CD24^hi^CD38^hi^ B cells from total CD19+ B cells by cytometry in a FacsVantage SE sorter (Beckton Dickinson). The purity of Breg-depleted B cells was >98%.

### B cell cultures

To assess the regulatory capacity of circulating CD19+CD24^hi^CD38^hi^ B cells (Breg) on B cell cytokine secretion, total CD19+ B cells or Breg-depleted CD19+ B cells (1x10^5^ cells/well) were cultured for 7 days in U-bottom 96-well plates containing RPMI 1640 medium (Lonza, Alendale, NJ, USA) with 10% FCS, 2 mM L-glutamine, 50 U/ml penicillin, 50 mg/ml streptomycin and 50 mM 2-mercaptoethanol, in the presence of 10μg/ml F(ab')2 Goat Anti-Human IgM+IgG Functional Grade Purified (eBioscience). Secretion of IFNγ was determined by ELISA in culture supernatants at different time points.

### Cell surface staining and flow cytometry

The frequency and phenotype of Breg cells present in the peripheral blood of AS patients and HC was assessed by flow cytometry after staining freshly isolated PBMCs with antibodies directed to surface phenotypical markers. Fluorochrome-conjugated mAbs from BD Pharmingen (San Diego, CA, USA) were used to examine the expression of CD3, CD4, CD19, CD24 and CD38 in a FACSCalibur flow cytometer with CellQuest software (BD Biosciences).

### ELISAs

Cell-free culture supernatants were collected and stored at -80°C. The concentration of IFNγ was determined by ELISA using a kit from BD Biosciences and following the manufacturer′s instructions. Serum calprotectin was determined by ELISA with a kit from Hycult Biotech (Ulden, The Netherlands).

### Statistical analysis

Comparison between groups was by Mann-Whitney test. Paired samples were compared using a Wilcoxon matched pairs signed rank sum test. Correlations were analyzed using Spearman’s rank correlation coefficients. P values less than 0.05 were considered significant. All analyses were performed using Prism version 5.0 software (GraphPad Software).

## Results

### Patients with AS/nb demonstrate increased numbers of circulating CD19+CD24^hi^CD38^hi^ B cells

**Fig 1:**
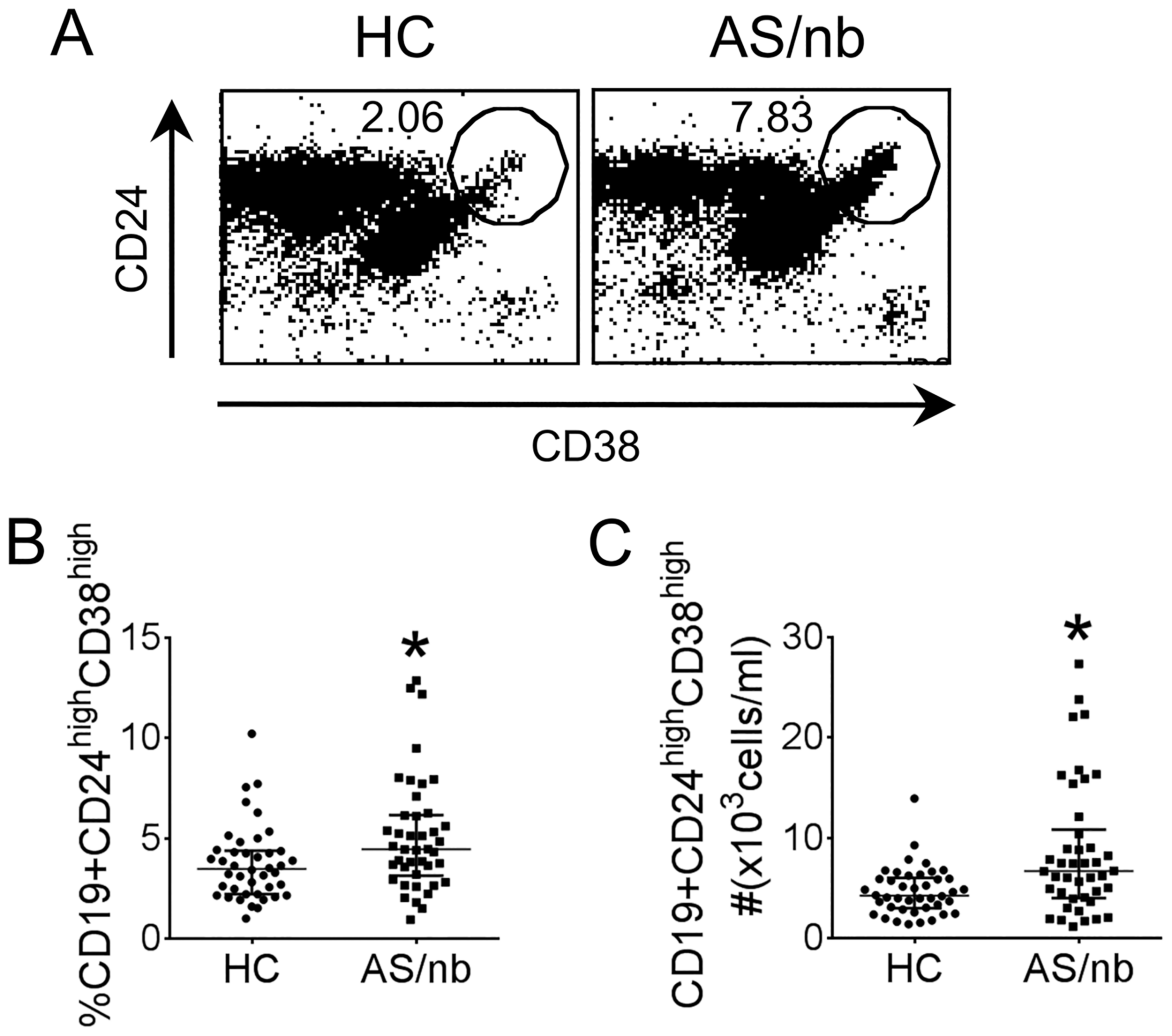
Numbers of circulating CD19+CD24^hi^CD38^hi^ B cells in AS patients naïve for TNF blockers (AS/nb). PBMCs isolated from the peripheral blood of AS/nb patients (n = 42) and from age and gender-matched healthy controls (HC) (n = 42) were stained with fluorochrome-labeled antibodies against cell surface markers and examined by flow cytometry. AS/nb patients showed an increased proportion and absolute numbers of circulating CD19+CD24^hi^CD38^hi^ B cells. A. Representative dot plots demonstrate CD24 and CD38 expression in cells gated for CD19. B, C. Frequency (B) and absolute numbers (C) of circulating CD19+CD24^hi^CD38^hi^ B cells in HC and AS/nb patients. Bars represent the median and interquartile range. *p<0.01.

We first sought to examine the expression of Breg phenotypical surface markers on CD19+ B cells present in the peripheral blood of AS/nb patients. The absolute numbers of circulating total CD19+ B cells were not different in AS/nb as compared with healthy control subjects: median (IQR), 151.7 (105.8–216.7) in AS/nb vs 131.6 (90.71–165.8) x10^3^/ml in HC, p>0.1. In contrast, both the frequency and the absolute number of circulating CD19+CD24^hi^CD38^hi^ B cells, were significantly increased in AS/nb patients naïve for TNF blockers, as compared with HC: median (IQR) 4.46% (3.08–6.20%) or 6.69 x10^3^/ml (3.98–11.24 x10^3^/ml) in AS/nb vs 3.28% (2.36–4.27%) or 4.17 x10^3^/ml (3.11–5.58 x10^3^/ml) in HC, p<0.01 (Fig 1).

### Relation of circulating CD19+CD24^hi^CD38^hi^ B cell frequency with disease activity

**Fig 2:**
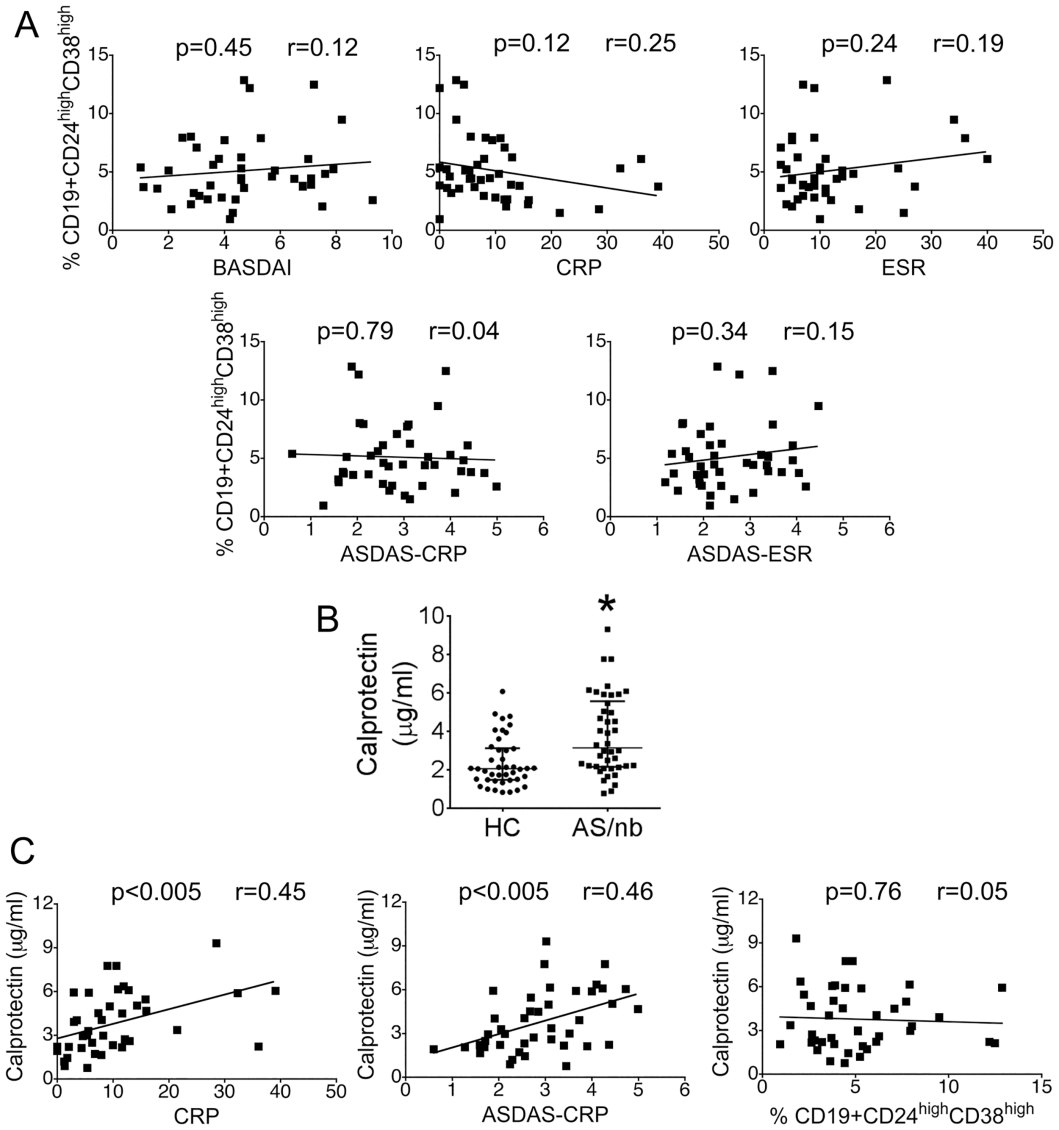
The frequency of circulating CD19+CD24^hi^CD38^hi^ B cells in AS/nb patients is not related with disease activity scores and/or inflammatory serological markers. A. The frequency of CD19+CD24^hi^CD38^hi^ B cells is not related with BASDAI, CRP, ESR, ASDAS-CRP or ASDAS-ESR. B. AS/nb patients demonstrate increased levels of calprotectin as compared with HC. *p <0.001 C. Serum calprotectin concentrations were significantly correlated with CRP and ASDAS-CRP but not with the frequency of CD19+CD24^hi^CD38^hi^ B cells.

The frequency of CD19+CD24^hi^CD38^hi^ B cells did not correlate significantly with BASDAI, CRP, ESR, ASDAS-CRP or ASDAS-ESR (Fig 2A). Patients with AS/nb demonstrated significantly elevated levels of serum calprotectin as compared with controls (p<0.001) (Fig 2B). Serum calprotectin correlated significantly with CRP and ASDAS-CRP but not with the frequency of circulating CD19+CD24^hi^CD38^hi^ B cells (Fig 2C).

### Treatment with anti-TNF agents is associated with a significant reduction of circulating CD19+CD24^hi^CD38^hi^ B cell numbers in AS patients

**Fig 3:**
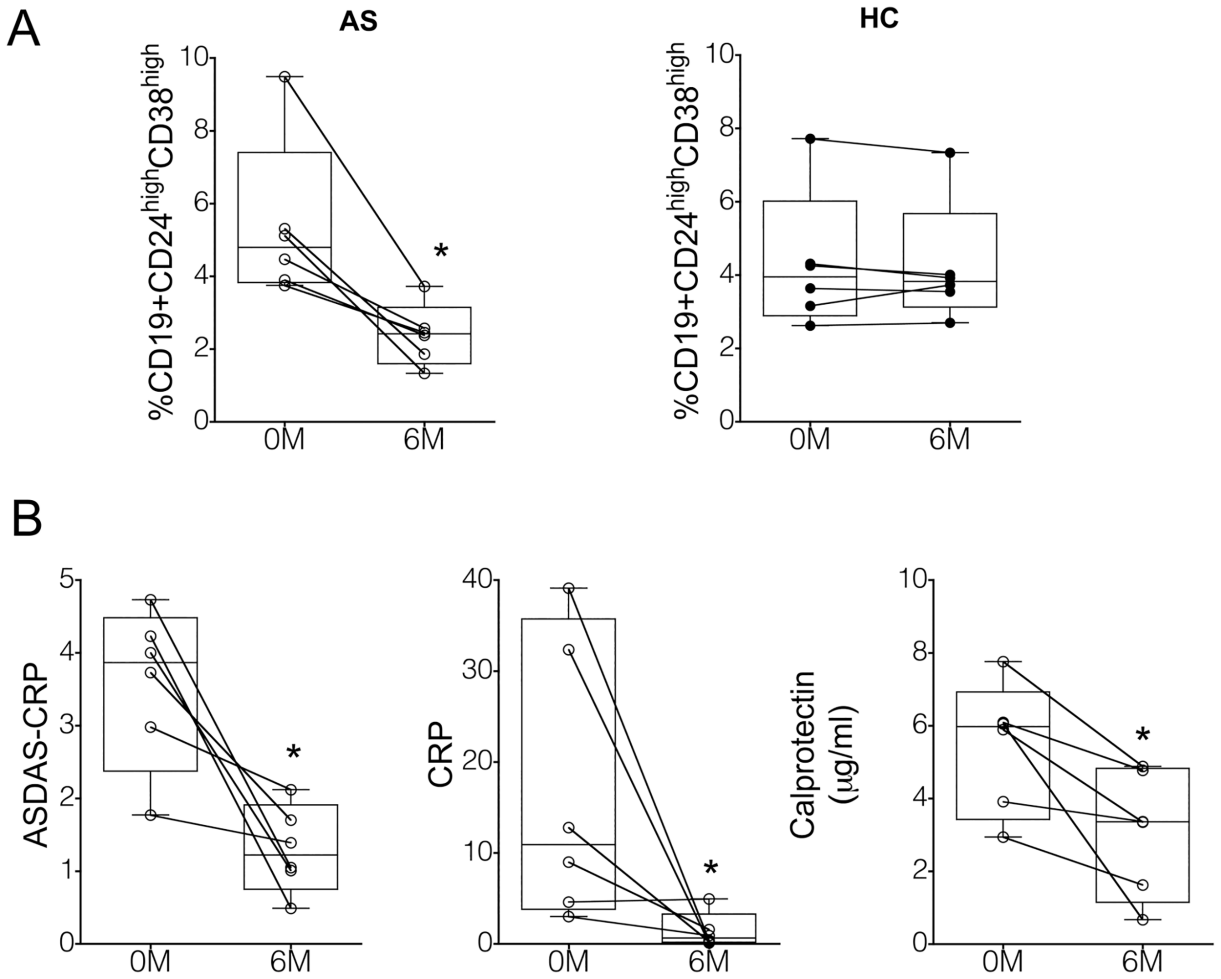
Treatment with anti-TNF agents is associated with a significant reduction of circulating CD19+CD24^hi^CD38^hi^ B cell numbers in AS patients. A. Frequency of CD19+CD24^hi^CD38^hi^ B cells in six AS patients before and six months after initiating treatment with anti-TNFα agents, and in their 6 age and gender-matched healthy controls at the basal and 6 month study. (*p<0.05) B. CRP, calprotectin and ASDAS-CRP values in these 6 AS/nb patients before and six months after initiating treatment with anti-TNFα. (*p<0.05)

We then sought to determine whether treatment with anti-TNFα agents is able to modify the altered numbers of Breg cells observed in the peripheral blood of AS/nb patients. Six AS patients previously naïve for TNF blockers and who had an incomplete response to NSAIDs, donated blood right before the initiation of an anti-TNFα monoclonal antibody, and six months thereafter. Blood was also obtained from six healthy subjects, who acted as controls for each patient at both the first and 6-month examination. When compared with basal data, at the six month evaluation all 6 AS patients demonstrated a marked reduction in the frequency of circulating Breg cells (p<0.05), whereas experimental variation observed in healthy controls was minimal (p>0.1) (Fig 3A). At the same time, decreased values of CRP (p<0.05), calprotectin (p<0.05) and ASDAS-CRP (p<0.05) were observed in all patients (Fig 3B). Four of these patients experienced a major clinical improvement as determined by a reduction of the ASDAS-CRP index greater than 2.0 units [16].

### Negative regulatory capacity of CD19+CD24^hi^CD38^hi^ B cells

**Fig 4:**
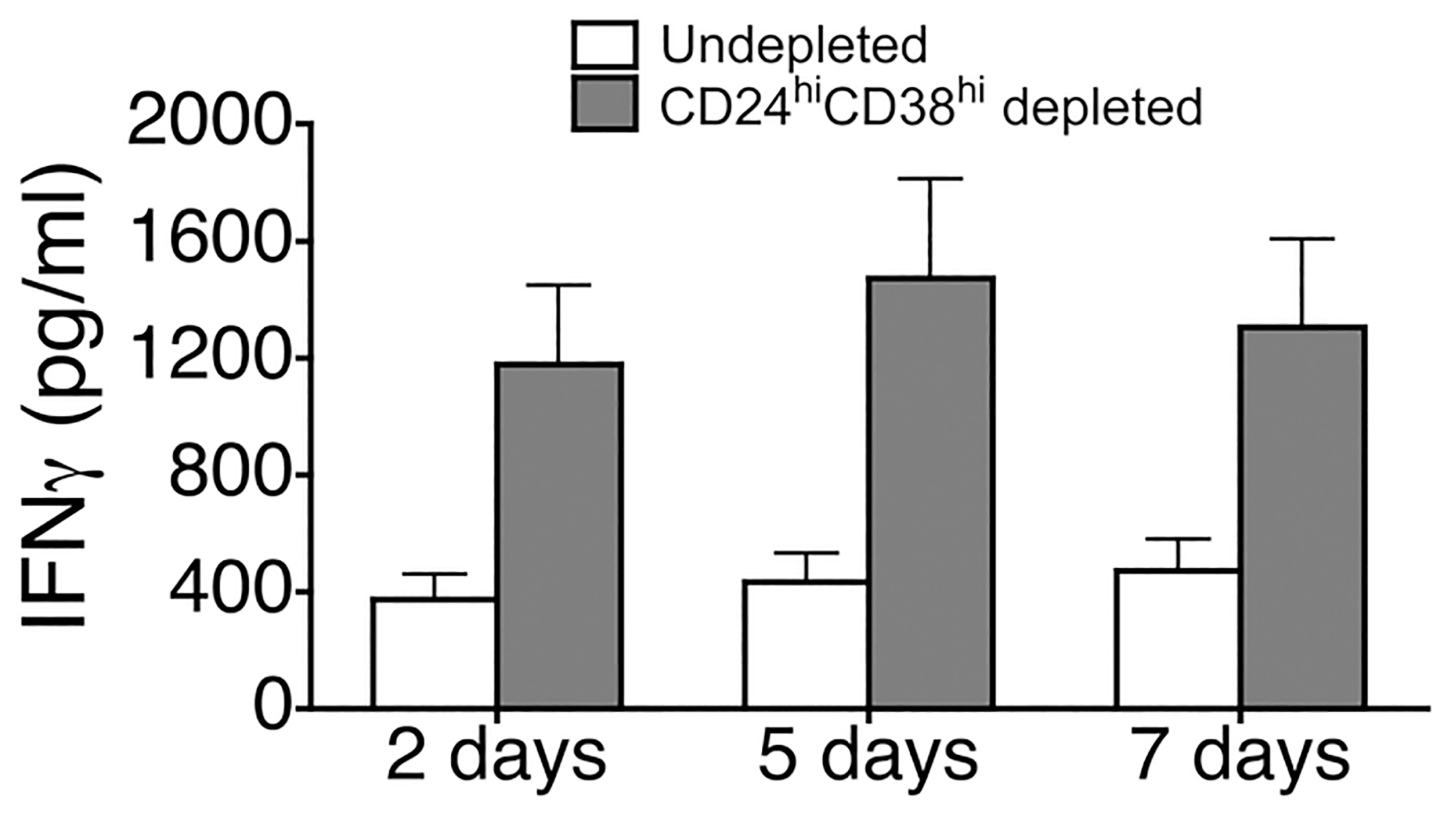
Suppressive capacity of CD19+CD24^hi^CD38^hi^ B cells in healthy subjects. Total CD19+ or B cells depleted of CD19+CD24^hi^CD38^hi^ cells (Breg-depleted CD19+ B cells), isolated from the peripheral blood of three healthy controls (HC), were established in culture, and stimulated with anti-IgM+IgG. Secretion of IFNγ was determined in culture supernatants by sandwich ELISA at 2, 5 or 7 days. Bar graphs represent the mean and SEM for IFNγ concentrations of 3 independent experiments.

Total CD19+ or Breg-depleted CD19+ B cells (B cells depleted of CD19+CD24^hi^CD38^hi^ cells) were isolated from the peripheral blood of three healthy controls (HC), established in culture and stimulated with anti-IgM+IgG MoAbs. Secretion of IFNγ was determined in culture supernatants by sandwich ELISA at 2, 5 or 7 days. When compared with total CD19+ B cells, Breg-depleted CD19+ B cells secreted an increased amount of IFNγ at all tested time points (2, 5 and 7 days) (Fig 4).

## Discussion

We have herein described an increased frequency and absolute number of CD19+CD24^hi^CD38^hi^ B cells in AS patients naïve for biological agents, that is not related with disease activity measures. These results parallel data published by Cantaert el al, who reported on elevated numbers of CD19+CD5+ B cells with a regulatory phenotype in SpA, independent of disease activity [2]. Assessment of clinical activity in SpA is difficult due to the lack of a universally accepted ‘gold standard’ [16], and because some of the currently used indices measure only one aspect of the disease or are mostly patient or physician oriented. Therefore we were interested in ascertaining whether the lack of correlation of Breg cell numbers with ASDAS-CRP was relevant. Serum calprotectin has recently emerged as a good biomarker for disease activity in SpA [17,18] and we observed that serum calprotectin concentrations were significantly correlated with CRP and ASDAS-CRP but not with the percentage of circulating Breg cells, thereby confirming that Breg cell numbers do not vary with disease activity in AS.

The gold standard for Breg cell definition remains the ascertainment of their regulatory capacity [6]. We observed that Breg-depleted CD19+ B cells secreted increased amounts of IFNγ as compared with total CD19+ B cells, indicating that Breg downregulate pro-inflammatory B cell cytokine secretion. Breg cells have been described to negatively modulate T cell responses [4–7] and downmodulate CD86 expression on B cells [8]. Our results indicate an autoregulatory action of Breg cells on B effector cell cytokine secretion, and this is an aspect of Breg function which had not been described previously.

The contribution of adaptive immunity to the pathogenesis of AS has not been established [1] and the possible role of increased Breg cells in the peripheral blood of AS patients does not have a straightforward explanation. One likely possibilty is that augmented Breg numbers are a reflection of chronic immune system stimulation at the gut. In fact, AS patients demonstrate an altered gut microbiome [19], which may be influenced by HLA-27 [20]. In addition, an increased intestinal permeability has been described not only in patients with AS but also in their healthy relatives [21]. Furthermore, subclinical gut inflammation can be observed in up to 65–70% of AS patients [22]. It is associated with an increased amount of local lymphoid follicles [23] and with a pro-angiogenic intestinal phenotype [24], that can facilitate an enhanced trafficking of immune cells.

Of note, the gut microbiota has been shown to be involved in the development of several autoimmune / autoinflammatory conditions including arthritis [25–27]. At the same time, it plays an important role in the generation of Breg cells thereby providing a negative feedback for inflammation [26,27]. Interestingly, the gut microflora is able to induce Breg differentiation not only at the intestinal lymphoid tissue, but also at the spleen [27]; this may indicate the existence of B cell trafficking from the gut to other organs [27], and/or the effect of specific gut microbiota metabolites signaling at distant sites [28].

In this context, there is evidence for an increased frequency of circulating CD19+/ CD38^hi^/ CD24^hi^/IL-10+ B cells in active IBD patients as compared with inactive disease or healthy controls [29], suggesting that Breg cells may act downregulating gut inflammation in patients with disease activity [29].

Importantly, whereas Breg numbers were significantly increased in our AS patients naïve for biological agents, independently of disease activity parameters, we observed that treatment with anti-TNFα was associated with a reduction of circulating Breg cell numbers. We can speculate that this is reflecting a modification of microbiome composition, gut barrier integritiy, and/or an improvement of subclinical gut mucosal inflammation that is only achieved by this treatment, given the leading role of TNFα in gut homeostasis and in the pathogenesis of inflammatory bowel disease [30–34]. Also, a potent downregulation of inflammatory mediators by anti-TNFα treatment may lead to reduced Breg numbers, given the dependency of Breg cell generation on the local inflammatory milieu [6].

This study is limited by the high amount of blood required for functional assays, which hampered the recruitement of patients for this purpose, and by the absence of clinical /laboratory data on subclinical gut inflammation or microbiota composition of our patients.

## Conclusion

In summary, we have described increased numbers of circulating CD19+CD24^hi^CD38^hi^ B cells (Breg) in AS/nb patients, that are not related with disease activity. Treatment with anti-TNFα drugs is able to downmodulate circulating Breg cell numbers in AS.

## Supporting information

S1 Table Clinical data of AS/nb patients. (DOCX)

S2 Table Age and gender of healthy controls. (DOCX)
