# Supplementary material for: Increased frequency of circulating CD19+CD24hiCD38hi B cells with regulatory capacity in patients with Ankylosing spondylitis (AS) naïve for biological agents
Source: PLoS One. 2017 Jul 6;12(7):e0180726. doi: 10.1371/journal.pone.0180726 (PMC5500370; doi:10.1371/journal.pone.0180726)
Supplement: S1 Table — (DOCX) [file pone.0180726.s001.docx]

**S1 table. Clinical data of AS/nb patients.**

|  | **Age (yrs) /Gender** | **HLA -B27** | **Symptoms duration**  **(years)** | **Time since diagnosis**  **(years)** | **BASDAI** | **BASFI** | **ASDAS -ESR** | **ASDAS -CRP** | **CRP** | **ESR** | **Treatment** | **Presen**  **tation** | **Other**  **manifestations** |
| --- | --- | --- | --- | --- | --- | --- | --- | --- | --- | --- | --- | --- | --- |
| **P 1** | 54/M | 1 | 36 | 35 | 7.6 | 6.9 | 3.92 | 4.28 | 10.6 | 16 | NSAID | Ax | - |
| **P 2** | 40/M | 1 | 21 | 17 | 2.1 | 0.4 | 2.15 | 3.02 | 28.5 | 17 | NSAID | Ax | - |
| **P 3** | 45/M | 1 | 32 | 25 | 4.4 | 3.5 | 2.38 | 3.40 | 11.6 | 6 | NSAID | Ax | - |
| **P 4** | 48/M | 1 | 24 | 3 | 7.9 | 0.3 | 2.23 | 2.29 | 1.25 | 4 | NSAID | Ax/P | - |
| **P 5** | 56/M | 1 | 30 | 11 | 7.5 | 7.1 | 3.07 | 4.10 | 11.9 | 5 | NSAID | Ax | - |
| **P 6** | 63/M | 1 | 34 | 34 | 1.1 | 0 | 1.36 | 1.71 | 6.25 | 8 | SSZ | Ax | Uveitis |
| **P 7** | 46/F | 0 | 19 | 16 | 4.2 | 5.2 | 2.14 | 1.27 | 0 | 10 | NSAID | Ax | - |
| **P 8** | 51/M | 1 | 36 | 27 | 3.4 | 2.2 | 1.96 | 2.75 | 12.1 | 6 | NSAID | Ax/P | - |
| **P 9** | 64/F | 1 | 46 | 15 | 4.7 | 1.5 | 2.30 | 1.88 | 2.95 | 22 | NSAID | Ax | - |
| **P 10** | 48/F | 1 | 16 | 8 | 4.9 | 7.6 | 2.77 | 2.03 | 0 | 9 | NSAID | Ax | - |
| **P 11** | 70/F | 0 | 13 | 8 | 3.8 | 1.8 | 2.14 | 2.55 | 7.95 | 11 | - | Ax | - |
| **P 12** | 47/M | 1 | 9 | 4 | 4.6 | 1.7 | 1.94 | 2.67 | 7.37 | 5 | SSZ | Ax/P | - |
| **P 13** | 74/F | 1 | 44 | 6 | 5.8 | 3.1 | 3.39 | 3.52 | 5.34 | 14 | NSAID | Ax | - |
| **P 14** | 35/F | 1 | 11 | 5 | 2.5 | 0 | 1.54 | 2.13 | 8.23 | 9 | NSAID | Ax | - |
| **P 15** | 56/F | 1 | 14 | 4 | 2.9 | 0.6 | 1.91 | 1.59 | 2.07 | 11 | - | Ax | - |
| **P 16** | 78/F | 1 | 19 | 22 | 1 | 4.1 | 1.32 | 0.60 | 0 | 10 | - | Ax | - |
| **P 17** | 57/M | 0 | 30 | 13 | 2.8 | 1.3 | 1.56 | 2.05 | 5.57 | 5 | SSZ | Ax | Uveitis |
| **P 18** | 58/F | 1 | 33 | 4 | 3.5 | 0.6 | 2.34 | 1.69 | 0 | 7 | NSAID | Ax | - |
| **P 19** | 25/M | 1 | 7 | 8 | 4 | 2.7 | 2.14 | 3.07 | 9.41 | 5 | NSAID | Ax | - |
| **P 20** | 25/F | 1 | 27 | 6 | 2.8 | 1.1 | 1.45 | 2.69 | 15.8 | 4 | - | Ax | - |
| **P 21** | 61/F | 1 | 47 | 12 | 4.3 | 5.5 | 2.66 | 3.13 | 21.5 | 25 | NSAID | Ax | - |
| **P 22** | 32/M | 1 | 19 | 16 | 3.9 | 0 | 1.92 | 2.55 | 9.98 | 9 | NSAID | Ax | - |
| **P 23** | 70/M | 1 | 53 | 3 | 1.6 | 0.4 | 1.86 | 1.91 | 3.5 | 11 | - | Ax | - |
| **P 24** | 67/F | 1 | 23 | 18 | 6.5 | 4.6 | 3.36 | 3.45 | 5.4 | 13 | MTX | Ax/P | - |
| **P 25** | 58/M | 1 | 42 | 42 | 4.7 | 1 | 2.01 | 2.24 | 1.33 | 3 | NSAID | Ax | - |
| **P 26** | 65/M | 1 | 24 | 23 | 3.6 | 1.2 | 1.62 | 2.44 | 6.74 | 3 | NSAID | Ax | - |
| **P 27** | 48/M | 1 | 6 | 5 | 3.1 | 0.7 | 1.18 | 1.60 | 7.89 | 7 | - | Ax | - |
| **P 28** | 34/F | 0 | 9 | 9 | 7 | 5.7 | 3.92 | 4.37 | 36.06 | 40 | - | Ax | - |
| **P 29** | 53/M | 1 | 35 | 14 | 7.2 | 6.7 | 3.48 | 3.90 | 4.35 | 7 | NSAID/SSZ | Ax | Uveitis |
| **P 30** | 55/M | 1 | 17 | 14 | 2 | 1.8 | 1.69 | 1.77 | 4.6 | 10 | NSAID/SSZ | Ax/P | - |
| **P 31** | 65/M | 1 | 37 | 35 | 5.3 | 2 | 3.49 | 3.10 | 10.9 | 36 | NSAID | Ax/P | - |
| **P 32** | 61/F | 1 | 20 | 2 | 6.8 | 5.1 | 3.68 | 4.44 | 14.3 | 9 | NSAID | Ax/P | - |
| **P 33** | 49/M | 0 | 10 | 2 | 9.3 | 9.1 | 4.20 | 4.99 | 15.96 | 12 | NSAID | Ax | - |
| **P 34** | 35/M | 0 | 10 | 10 | 4.6 | 4.7 | 2.23 | 2.98 | 9.01 | 9 | NSAID | Ax | - |
| **P 35** | 43/F | 1 | 3 | 2 | 8.2 | 9.5 | 4.47 | 3.73 | 3 | 34 | NSAID | Ax | - |
| **P 36** | 39/M | 1 | 1 | 0 | 7.1 | 6.5 | 3.39 | 4.23 | 12.8 | 7 | NSAID | Ax/P | - |
| **P 37** | 31/M | 1 | 8 | 1 | 4.6 | 0.7 | 3.24 | 4.00 | 32.34 | 24 | MTX/SSZ | Ax/P | - |
| **P 38** | 64/F | 1 | 22 | 10 | 6.8 | 5.7 | 4.05 | 4.73 | 39.1 | 27 | NSAID | Ax/P | - |
| **P 39** | 30/M | 1 | 10 | 10 | 7.1 | 4.3 | 3.07 | 3.66 | 5.65 | 5 | MTX | Ax | Uveitis |
| **P 40** | 51/M | 1 | 20 | 20 | 3 | 4.6 | 1.94 | 2.85 | 11.62 | 3 | NSAID | Ax | - |
| **P 41** | 61/M | 1 | 40 | 24 | 4.6 | 7.5 | 2.39 | 3.13 | 12.96 | 6 | NSAID | Ax | - |
| **P 42** | 39/M | 0 | 4 | 3 | 5.7 | 7.9 | 2.93 | 2.56 | 1.8 | 14 | SSZ | Ax/P | - |

P1: patient 1; P2: Patient 2 ... Pn : Patient n; F: female; M: male; Ax: axial; P: peripheral; Ax/P: axial and peripheral
