# Supplementary material for: Increased frequency of circulating CD19+CD24hiCD38hi B cells with regulatory capacity in patients with Ankylosing spondylitis (AS) naïve for biological agents
Source: PLoS One. 2017 Jul 6;12(7):e0180726. doi: 10.1371/journal.pone.0180726 (PMC5500370; doi:10.1371/journal.pone.0180726)
Supplement: S2 Table — (DOCX) [file pone.0180726.s002.docx]

**S2 table. Age and gender of healthy controls.**

|  | **Age (yrs)/ gender** |
| --- | --- |
| **HC 1** | 47/M |
| **HC 2** | 33/M |
| **HC 3** | 48/M |
| **HC 4** | 51/M |
| **HC 5** | 60/M |
| **HC 6** | 63/M |
| **HC 7** | 32/F |
| **HC 8** | 56/M |
| **HC 9** | 59/F |
| **HC 10** | 53/F |
| **HC 11** | 62/F |
| **HC 12** | 55/M |
| **HC 13** | 63/F |
| **HC 14** | 32/F |
| **HC 15** | 51/F |
| **HC 16** | 62/F |
| **HC 17** | 56/M |
| **HC 18** | 57/F |
| **HC 19** | 31/M |
| **HC 20** | 27/F |
| **HC 21** | 59/F |
| **HC 22** | 35/M |
| **HC 23** | 56/M |
| **HC 24** | 63/F |
| **HC 25** | 56/M |
| **HC 26** | 47/M |
| **HC 27** | 30/M |
| **HC 28** | 35/F |
| **HC 29** | 58/M |
| **HC 30** | 51/M |
| **HC 31** | 64/F |
| **HC 32** | 56/F |
| **HC 33** | 43/M |
| **HC 34** | 31/M |
| **HC 35** | 50/F |
| **HC 36** | 41/M |
| **HC 37** | 36/M |
| **HC 38** | 61/F |
| **HC 39** | 33/M |
| **HC 40** | 47/M |
| **HC 41** | 58/M |
| **HC 42** | 37/M |

HC: Healthy control; F: female; M: male
